# Short-Term Analysis of the Changes in the Bone Mineral Density of the Proximal Femur After Uncemented Total Hip Arthroplasty: A Prospective Study of 110 Patients

**DOI:** 10.7759/cureus.23257

**Published:** 2022-03-17

**Authors:** Shubhranshu S Mohanty, Akash N Vasavda, Abhishek K Rai, Tushar N Rathod, Prashant Kamble, Swapnil Keny

**Affiliations:** 1 Department of Orthopaedics, Seth Gordhandas Sunderdas Medical College (GSMC) and King Edward Memorial (KEM) Hospital, Mumbai, IND

**Keywords:** gruen zones, dexa scan, uncemented tha, total hip replacement, bone mineral density

## Abstract

Introduction

Mechanical loading continuously exposes the bone to remodeling processes. Increased load leads to a gain in bone mass, and reduced load results in a loss. After inserting a prosthesis, the proximal femur is bypassed in loading as the bodyweight shifts distally. This lack of load induces bone resorption according to Wolff’s law. To avoid this bone resorption, the implant's bending stiffness should be less than the femoral bone. Dual-energy X-ray absorptiometry (DEXA) is a well-accepted method to measure periprosthetic bone mineral density (BMD) after total hip arthroplasty (THA). Since the strength and durability of the fixation of a femoral prosthesis in cementless hip arthroplasty depend largely on the quantity and quality of the surrounding bone, preoperative and postoperative evaluation of the quantity and quality of the femoral bone is very important in the long-term prognosis of hip arthroplasty.

Materials and methods

A prospective study of 110 patients in the age group of 25-60 years who underwent uncemented total hip arthroplasty in our tertiary healthcare institution was performed. An uncemented, fully hydroxyapatite-coated implant from a single manufacturer was used in all the patients. All the patients were mobilized on the first post-operative day. The BMD was measured at the proximal femur and the distal tip using a DEXA scan. Gruen zones were used for calculating BMD at different anatomical locations in the femur, with particular importance to zones 1, 4, and 7.

Results

The pre-operative BMD in all zones measured, viz. zones 1, 4, and zone 7 of the affected side, was found to be significantly lower as compared to the BMD values on the control side (P< 0.05). The mean change in the mean BMD was calculated for all the zones and compared with each other using an unpaired t-test. The mean BMD changes were found to be significantly higher in zone 7 in comparison to both zones 1 and 4 (p<0.05).

Conclusion

Significant periprosthetic bone loss after uncemented THA in the femur was noted in Gruen zones 1, 4, and 7 during the first six months after THA, with the greatest bone loss in the femoral calcar area (zone 7). The lower the preoperative BMD of the patient, the greater the postoperative bone loss.

## Introduction

The primary choice for implant fixation in osteoporosis for arthroplasty in recent times has been uncemented total hip arthroplasty (THA), but its long-term durability and stability may be limited by progressive bone loss around the implant [1]. Mechanical loading continuously exposes the bone to remodelling processes. Increased load results in a gain of bone mass, and reduced load results in a loss. The implantation of a THA prosthesis changes the distribution of mechanical forces around the hip joint [2]. After inserting a prosthesis, the proximal femur is bypassed during loading as the bodyweight is transferred distally. This lack of loading induces bone resorption according to Wolff’s law. Periprosthetic bone resorption is more pronounced with large stems and stems with an extensive porous coating [3].

Several studies have shown that low bone mineral density (BMD) in the hip affects the longevity of prosthetic implants following total hip arthroplasty [4]. Low bone mineral density in combination with a high body mass index increases the risk of early femoral component failure following hip arthroplasty [5]. Periprosthetic bone loss is proportional to the pre-operative bone mineral density in the periprosthetic Gruen zones, which leads to implant loosening. As the strength and durability of the fixation of a femoral prosthesis in uncemented hip arthroplasty depend largely on the quantity and quality of the surrounding bone, preoperative and postoperative evaluation of the same is very important in prognosticating the long-term survival of hip arthroplasty. Serial radiographs, dual-energy X-ray absorptiometry (DEXA), and quantitative CT have been used to evaluate bone density changes [6]. As the radiographs suffer from high interobserver variability, the DEXA scan is the preferred method for the assessment of changes in bone mass around prostheses.

## Materials and methods

A prospective study of 110 patients in the age group of 25-60 years who underwent uncemented total hip arthroplasty in our tertiary healthcare institution was performed after obtaining institutional ethics committee approval (IRB Approval number: EC/24/2019). The selection criteria included patients with unilateral avascular necrosis of the femoral head. Patients with pre-existing metabolic disorders such as Paget’s disease, parathyroid gland disorders, patients taking drugs such as corticosteroids and estrogens, and patients with bilateral avascular necrosis of the femoral head were excluded. Also, all non-ambulatory patients and those in whom an intraoperative complication resulted in delayed mobilization were excluded.

**Figure 1:**
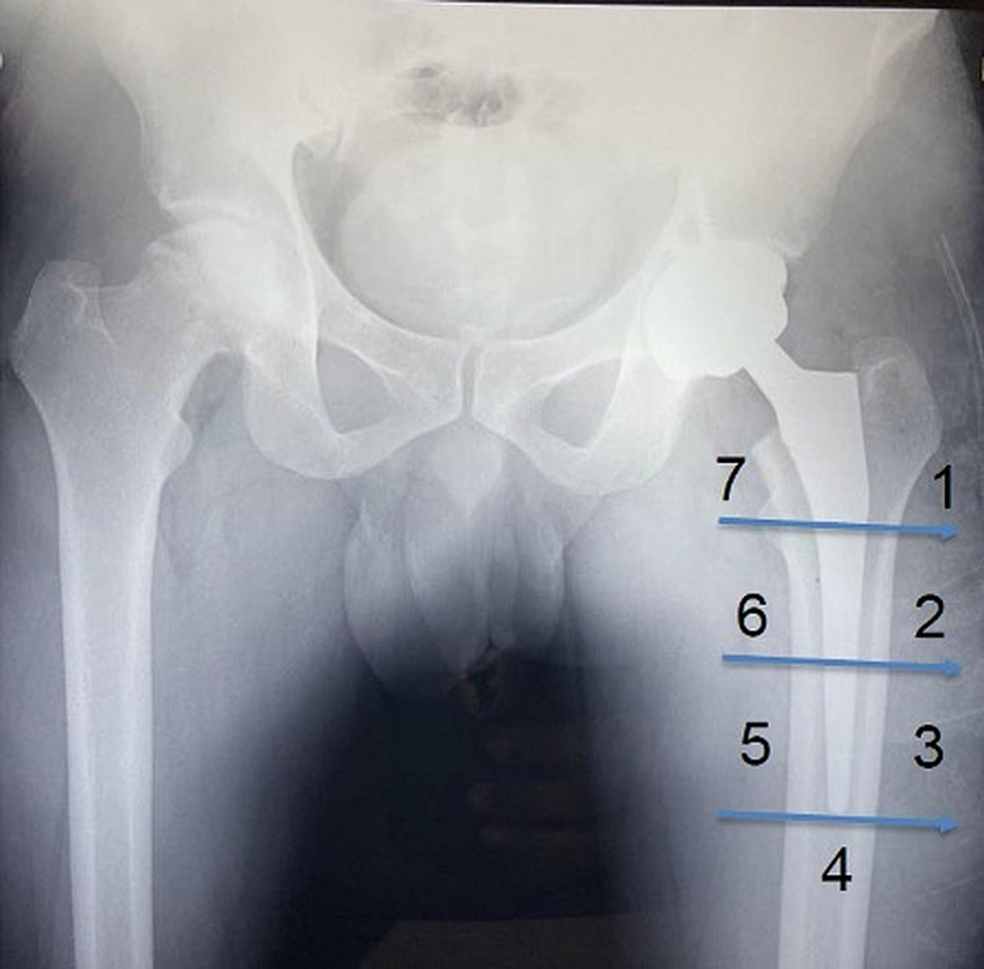
Illustration of various Gruen zones in the proximal femur.

The data collected included demographic details, presence of co-morbidities, preoperative BMD and BMD at six months' follow-up. The BMD was measured using a DEXA scan with the patient in a supine position and the femur in neutral rotation with standard knee and foot supports. The values were expressed as BMD in gram/centimeter^3^. All DEXA measurements were performed using the same apparatus. BMD at the proximal femur and the distal tip of the prosthesis was measured. For ease of measurement, although described for cemented hips, Gruen zones were used in our study for calculating BMD at different anatomical locations in the femur. Of particular importance were zones 1, 4, and 7 without the inclusion of metal prostheses (Figure 1). Apart from this, the BMD of the opposite hip was also calculated. For comparison, the unaffected opposite side was kept as the control side. The patients were followed up at six months post-operatively for a repeat DEXA scan to analyse the changes in the BMD on both the affected and unaffected sides and to compare these changes. All patients were operated on by the senior-most author through a standard posterolateral approach without removal of the greater trochanter. An uncemented, fully hydroxyapatite-coated implant from a single manufacturer was used in all the patients. The patients were mobilized on the first post-operative day.

Data analysis

The data were collected, compiled, and analysed. The descriptive data (mean and standard deviation) were calculated for continuous variables. Frequencies and percentages were calculated for categorical variables. The association between variables was analysed by the chi-square test for categorical variables. An unpaired t-test was used to compare the mean of quantitative variables between the two study groups.

## Results

A total of 110 patients were included in this prospective study. The majority of the patients in the study were males (68 patients, 62%). The mean age of the patients was found to be 49.4 years (range: 25-60 years). Fifty-seven of the 110 patients (52%) in the study belonged to the age group of 51-60 years. Patients having unilateral avascular necrosis of the femoral head were included in the study. The normal, unaffected side in the unilateral cases was kept as a control. 

The mean BMD of all the patients was measured pre-operatively for both the affected and the control side. The mean pre-operative BMD in all zones measured, viz. zones 1, 4, and 7 of the affected side, was found to be significantly lower as compared to the BMD values on the control side (p<0.05; Table 1).

**Table 1:** Pre-operative mean BMD of various zones on affected and the control side. *Values are significant, p<0.01. BMD: bone mineral density.

Zones	Affected side BMD values (n=110)	Control side BMD values (n=110)	P-value
Zone 1	1.61* ± 0.15	1.66 ± 0.15	<0.01
Zone 4	1.55* ± 0.13	1.63 ± 0.13	<0.01
Zone 7	1.49* ± 0.14	1.58 ± 0.14	<0.01

The mean BMD was calculated at a follow-up of six months in all the patients. In all zones measured, viz. zones 1, 4, and 7, the mean BMD of the affected side was found to be significantly lower as compared to the BMD values on the control side at six months of follow-up (p<0.05; Table 2). The mean BMD of all the zones on the control side was the same as the pre-operative values.

**Table 2:** Mean BMD of various zones at post-operative follow up of six months on affected and the control side. *Values are significant, p<0.01. BMD: bone mineral density.

Zones	Affected side BMD values (n=110)	Control side BMD values (n=110)	P-value
Zone 1	1.51* ± 0.12	1.66 ± 0.15	<0.01
Zone 4	1.47* ± 0.14	1.63 ± 0.13	<0.01
Zone 7	1.32* ± 0.14	1.58 ±0.14	<0.01

On comparing the mean BMD of the affected side at six months of follow-up to the preoperative values, mean BMD in all zones of the affected side, viz. zones 1, 4, and 7, was found to be significantly lower as compared to the BMD values on the affected side preoperatively (p<0.05; Table 3).

**Table 3:** Comparison of the pre-operative mean BMD with the post-operative values on the affected side. *Values are significant, p<0.01. BMD: bone mineral density.

Zones	Preoperative values (BMD)	Six-month follow-up values (BMD)	P-value
Zone 1	1.61 ± 0.15	1.51 ± 0.14	<0.01
Zone 4	1.55 ± 0.13	1.47 ± 0.14	<0.01
Zone 7	1.49 ± 0.14	1.32 ± 0.14	<0.01

The change at six-month follow-up in the mean BMD was calculated for all the zones on the affected side and compared with the pre-operative values of the same zones using an unpaired t-test. The mean BMD changes were found to be significantly higher in zone 7 in comparison to both zones 1 and 4 (p<0.05; Tables 4-5).

**Table 4:** Mean change in the BMD of various zones at follow up of six months on the affected side. BMD: bone mineral density.

Zones	Mean change in BMD (g/cm^3^)
Zone 1	0.09 ± 0.06
Zone 4	0.08 ± 0.05
Zone 7	0.17 ± 0.15

**Table 5:** Comparison between mean change in BMD in different zones at follow up on affected side (P-values). *Values are significant, p<0.01. BMD: bone mineral density.

Zone 1 vs zone 4	Zone 1 vs zone 7	Zone 4 vs zone 7
0.71	<0.001*	<0.001*

## Discussion

Stress shielding due to distal load transfer between the prosthesis and the surrounding host bone is a common phenomenon on the femoral side after THA. DEXA is a fast and reliable method for the quantification of bone mass in several skeletal regions. The recent development of analysis programs with a metal reduction option permitted the evaluation of periprosthetic bone density and suggested a potential application of DEXA in the field of THA. In this study, the change in periprosthetic bone density after uncemented THA was determined [7]. The rapid circumferential decrease in periprosthetic BMD early after surgery is considered to be a result of intramedullary circulation disorders and endosteal injury associated with the surgical manoeuvres and temporary enhancement of bone turnover due to postoperative immobilization, lack of weight-bearing, and disuse. The literature review of similar studies done in the past showed a marked fall in BMD in all Gruen zones in the first postoperative year. Since the most statistically significant difference was found in zones 1 and 7, we decided to study these areas in greater detail in our study [8]. In view of certain reports of raised BMD postoperatively in Gruen zone 4, we included this intermediate zone also in our study.

In our study, there was a reduction in the mean preoperative BMD across all the zones, viz. zones 1, 4, and 7, at six-month follow-up as compared to pre-operative values. The most significant change in BMD was seen in zone 7. We also compared the unaffected control side, which showed no difference between pre-and post-operative readings. The study of Venesmaa et al. on 22 patients corroborates with our study [9]. They studied changes in bone mass after uncemented THA. In their study, there was a significant reduction in the mean BMD of various Gruen zones at one year following compared with pre-operative values. The reduction in the mean BMD values was most significant in zone 7.

Brodner et al. studied the bone mineral density of the proximal femur after implantation of a tapered rectangular cementless stem in 100 patients with a mean age of 60 years in all the Gruen zones [10]. They performed a DEXA scan one week after surgery and then every six months until the end of five years. The BMD increased significantly in Gruen zones 2, 4, and 5, and decreased significantly in Gruen zones 1, 6, and 7 over the five-year follow-up. The decrease in BMD was seen for the first 12 months after surgery. The study observed that there was a gradual rise in the BMD after a period of one year. At the end of five years, the mean BMD values across all zones were similar to the pre-operative ones. Our findings are in concordance with this study stating the point that maximum reduction in the post-operative BMD was observed in zone 7. Kiratli et al. [11] performed a short-term longitudinal evaluation on 35 patients with one year of follow-up and reported that BMD decreased in all periprosthetic zones beginning immediately after surgery and that the decrease was rapid in the first three months but slowed thereafter, achieving equilibrium at six months after surgery. Similar results were found in our study, which showed a significant reduction in BMD across all zones for the first six months.

In the longitudinal analysis, various studies analysed the changes in femoral BMD after hip arthroplasty by comparing them with the value one month after surgery to eliminate the effects of morphological changes in the femoral cortico-endosteal surface associated with the surgical manoeuvre. [12] In our study, the femoral BMD of the normal side was used as the control, following the methods proposed by various investigators to correct for individual variation in femoral BMD. In our study, we have studied pre- and post-operative BMD to eliminate the effects of morphological changes in the femur cortico-endosteal surface associated with the surgical manoeuvre. As a result, the changes observed on cross-sectional analysis were greater than the actual postoperative changes observed on longitudinal analysis. Therefore, the changes in BMD obtained by cross-sectional analysis cannot be regarded as equivalent to those observed by longitudinal analysis. However, cross-sectional analysis is considered useful for the evaluation of the pattern of short-term to mid-term postoperative changes in periprosthetic BMD of the femur. We carried out a prospective study that accurately showed the bone response to a metallic implant in the femur after uncemented THA. Preoperative BMD measurements can be used to identify patients with low bone mass. The study points out that there is a need for study on early BMD and its prevention by supplementing the patients with osteoinductive medications, which may improve the longevity and durability of the femoral prosthesis.

There are a few limitations to this study. The sample size is small enough to detect clinically significant differences in bone loss between different stem sizes. Of all the similar studies available in the literature, our study has the highest sample size. The follow-up time was too short to draw conclusions about the long-term longevity of uncemented THA. A study with a larger sample size and long-term follow-up is needed to enable us to incorporate these findings into clinical use.

## Conclusions

Significant periprosthetic bone loss after uncemented total hip arthroplasty in the femur was noted in Gruen zones 1, 4, and 7 during the first six months after THA, with the greatest bone loss in the femoral calcar area (zone 7). This study emphasizes the fact that there needs to be a study on the efficacy of various pre-operative and post-operative osteoinductive medications that may prevent the decrease in bone mineral density.
